# Nutraceuticals and COVID‐19: A mechanistic approach toward attenuating the disease complications

**DOI:** 10.1111/jfbc.14445

**Published:** 2022-10-14

**Authors:** Keshav Raj Paudel, Vyoma Patel, Sukriti Vishwas, Saurabh Gupta, Sumit Sharma, Yinghan Chan, Niraj Kumar Jha, Jesus Shrestha, Mohammad Imran, Nisha Panth, Shakti Dhar Shukla, Saurav Kumar Jha, Hari Prasad Devkota, Majid Ebrahimi Warkiani, Sachin Kumar Singh, Md Khadem Ali, Gaurav Gupta, Dinesh Kumar Chellappan, Philip M. Hansbro, Kamal Dua

**Affiliations:** ^1^ Centre of Inflammation, Centenary Institute and University of Technology Sydney, Faculty of Science School of Life Sciences Sydney Australia; ^2^ Discipline of Pharmacy, Graduate School of Health University of Technology Sydney Sydney New South Wales Australia; ^3^ Faculty of Health, Australian Research Centre in Complementary and Integrative Medicine University of Technology Sydney Ultimo New South Wales Australia; ^4^ School of Clinical Medicine, Faculty of Medicine and Health University of New South Wales Sydney New South Wales Australia; ^5^ School of Pharmaceutical Sciences Lovely Professional University Phagwara India; ^6^ Delhi Pharmaceutical Sciences and Research University New Delhi India; ^7^ Department of Life Sciences, School of Pharmacy International Medical University Kuala Lumpur Malaysia; ^8^ Department of Biotechnology, School of Engineering & Technology (SET) Sharda University Greater Nioda India; ^9^ School of Biomedical Engineering University of Technology Sydney Sydney New South Wales Australia; ^10^ Therapeutics Research Group, The University of Queensland Diamantina Institute, Faculty of Medicine University of Queensland Brisbane Queensland Australia; ^11^ Department of Biomedicine, Health and Life Convergence Sciences, Biomedical and Healthcare Research Institute Mokpo National University Muan Korea; ^12^ Graduate School of Pharmaceutical Sciences Kumamoto University Kumamoto Japan; ^13^ Institute for Biomedical Materials and Devices, Faculty of Science University of Technology Sydney Sydney New South Wales Australia; ^14^ Department of Medicine, Division of Pulmonary, Allergy and Critical Care Medicine Stanford University Stanford California USA; ^15^ Vera Moulton Wall Center for Pulmonary Vascular Disease Stanford University Stanford California USA; ^16^ School of Pharmacy Suresh Gyan Vihar University Jaipur India; ^17^ Department of Pharmacology, Saveetha Dental College and Hospitals, Saveetha Institute of Medical and Technical Sciences Saveetha University Chennai India; ^18^ Uttaranchal Institute of Pharmaceutical Sciences Uttaranchal University Dehradun India

**Keywords:** COVID‐19, nutraceuticals, probiotics, vitamins

## Abstract

Nutraceuticals have emerged as potential compounds to attenuate the COVID‐19 complications. Precisely, these food additives strengthen the overall COVID treatment and enhance the immunity of a person. Such compounds have been used at a large scale, in almost every household due to their better affordability and easy access. Therefore, current research is focused on developing newer advanced formulations from potential drug candidates including nutraceuticals with desirable properties viz, affordability, ease of availability, ease of administration, stability under room temperature, and potentially longer shelf‐lives. As such, various nutraceutical‐based products such as compounds could be promising agents for effectively managing COVID‐19 symptoms and complications. Most importantly, regular consumption of such nutraceuticals has been shown to boost the immune system and prevent viral infections. Nutraceuticals such as vitamins, amino acids, flavonoids like curcumin, and probiotics have been studied for their role in the prevention of COVID‐19 symptoms such as fever, pain, malaise, and dry cough. In this review, we have critically reviewed the potential of various nutraceutical‐based therapeutics for the management of COVID‐19. We searched the information relevant to our topic from search engines such as PubMed and Scopus using COVID‐19, nutraceuticals, probiotics, and vitamins as a keyword. Any scientific literature published in a language other than English was excluded.

**Practical applications:**

Nutraceuticals possess both nutritional values and medicinal properties. They can aid in the prevention and treatment of diseases, as well as promote physical health and the immune system, normalizing body functions, and improving longevity. Recently, nutraceuticals such as probiotics, vitamins, polyunsaturated fatty acids, trace minerals, and medicinal plants have attracted considerable attention and are widely regarded as potential alternatives to current therapeutic options for the effective management of various diseases, including COVID‐19.

## Abstract 2

Regular consumption of such nutraceuticals has been shown to boost the immune system and prevent viral infections. Nutraceuticals such as vitamins, amino acids, flavonoids like curcumin, and probiotics have been studied for their beneficial effects in the prevention of COVID‐19 symptoms such as fever, pain, malaise, and dry cough. As such, various nutraceutical‐based products such as compounds could be promising agents for the effective management of COVID‐19 symptoms and complications.

## 1 INTRODUCTION

The coronavirus pandemic that emerged during the latter part of December 2019 continues to cause a massive health risk to the global population. Citizens from various countries have been provided free COVID‐19 vaccines as it is a highly contagious respiratory disease caused by a novel pathogen belonging to the family *Coronaviridae*. The causative pathogen was initially named novel coronavirus 2019 (2019‐nCoV) by the WHO, which was then renamed severe acute respiratory syndrome (SARS) coronavirus‐2 (SARS‐CoV)‐2 as genomic studies had by then revealed that it was highly homologous to SARS‐CoV, which was the pathogen responsible for the significant outbreak of the SARS pandemic during the years 2002–2003 (Mehta et al., 2020; Parasher, 2021). Generally, individuals infected with SARS‐CoV‐2 present early symptoms of fever, dry cough, shortness of breath, and tachypnoea. Other reported symptoms include malaise, muscle pain, weakness, respiratory distress, as well as loss of taste and smell. Recovery from mild infection of SARS‐CoV‐2 commonly occurs within seven to ten days upon onset of symptoms, whereas it could take up to three to six weeks in severe and critical illness (Esakandari et al., 2020; Raveendran et al., 2021). Nevertheless, there is a subset of patients who even after recovery continue to experience various symptoms that persist and do not resolve over the course of many months to years. These patients are classified as having long COVID or post‐acute sequelae of COVID‐19 (PASC) (Proal & VanElzakker, 2021). The clinical manifestations and symptoms of PASC are clinically heterogenous which suggests the involvement of multiple organs and systems. Fatigue, headache, myalgia, loss of memory, breathlessness, and cognitive impairment are among the prevalent symptoms of PASC. Hence, both COVID‐19 and PASC represent a major burden to public healthcare systems while contributing to substantial economic and productivity losses, especially in low‐ and middle‐income countries. Given the high infectivity rate of SARS‐CoV‐2, the identification and development of novel agents for the treatment of COVID‐19 is crucial to reducing its impact on global morbidity and mortality.

Nutraceuticals are hybrid products generally containing both nutrients and pharmaceuticals. In addition to their medicinal properties, they possess nutritional values, acting as dietary supplements for maintaining body health, at the same time, providing the body with the necessary nutrition that is required for multiple metabolic processes and the regulation of normal body functions. Typically, the use of nutraceuticals can aid in the prevention and treatment of diseases, as well as promote physical health and the immune system, normalizing body functions, and improving longevity (Chan et al., 2021; Chanda et al., 2019). Nutraceuticals also present several advantages in contrast to synthetic drugs. For instance, the use of nutraceuticals can reduce the high production costs of modern pharmaceuticals. The costs involved in setting up a mass production system for growing plants as well as the maintenance of infrastructure involved in the harvesting of natural products and curation of raw extracts are remarkably lower. Therefore, deploying such a system in low‐ to middle‐income countries in which high infrastructure costs represent a barrier to research and development of novel therapeutics is highly feasible. Moreover, such low production costs can effectively lower the price of therapeutics to be made more affordable, thereby addressing the issue of inequality in access to medicines, especially for patients residing in rural areas in low‐income countries (Sack et al., 2015; Sofowora et al., 2013). Therefore, a gap between different therapies to combat the infections with COVID‐19 could be reduced to a maximum extent by the addition of nutraceuticals to the diet. Also, due to their significant positive outcomes, these compounds improve overall human health and thus, the application of nutraceuticals has emerged as an option to prevent comorbidity. Nutraceuticals are typically derived from natural sources, they could overcome the limitations of poor tolerability and toxicity, as well as the poor safety profile that is often associated with synthetic compounds (Gulati et al., 2016; Imran, Saleem et al., 2020). Studies have also shown that natural products imbue physicochemical characteristics superior to synthetic compounds such as fewer aromatic rings, lower nitrogen content, and increased oxygen content, thereby contributing to their greater ability to actively engage biological targets and produce biological activity (Wright, 2019). Hence, nutraceuticals such as probiotics, vitamins, polyunsaturated fatty acids, trace minerals, and medicinal plants have attracted considerable attention from medical researchers, and they are widely regarded as potential alternatives to current therapeutic options for the effective management of various diseases, including COVID‐19.

## 2 INTRODUCTION TO NUTRACEUTICALS

Nutraceuticals are well‐known compounds in the form of nutrients and pharmaceuticals that provides benefits in terms of medical and health by preventing and treating any disease. Previously, several publications demonstrated the role of nutraceuticals in the management of COVID‐19. A detailed discussion on the role of nutraceuticals in improving human health and attenuating disease complications has been mentioned in the next sections of the manuscript.

## 3 NUTRACEUTICALS AND THE MANAGEMENT OF COVID

### 3.1 Management of acute symptoms

**FIGURE 1:**
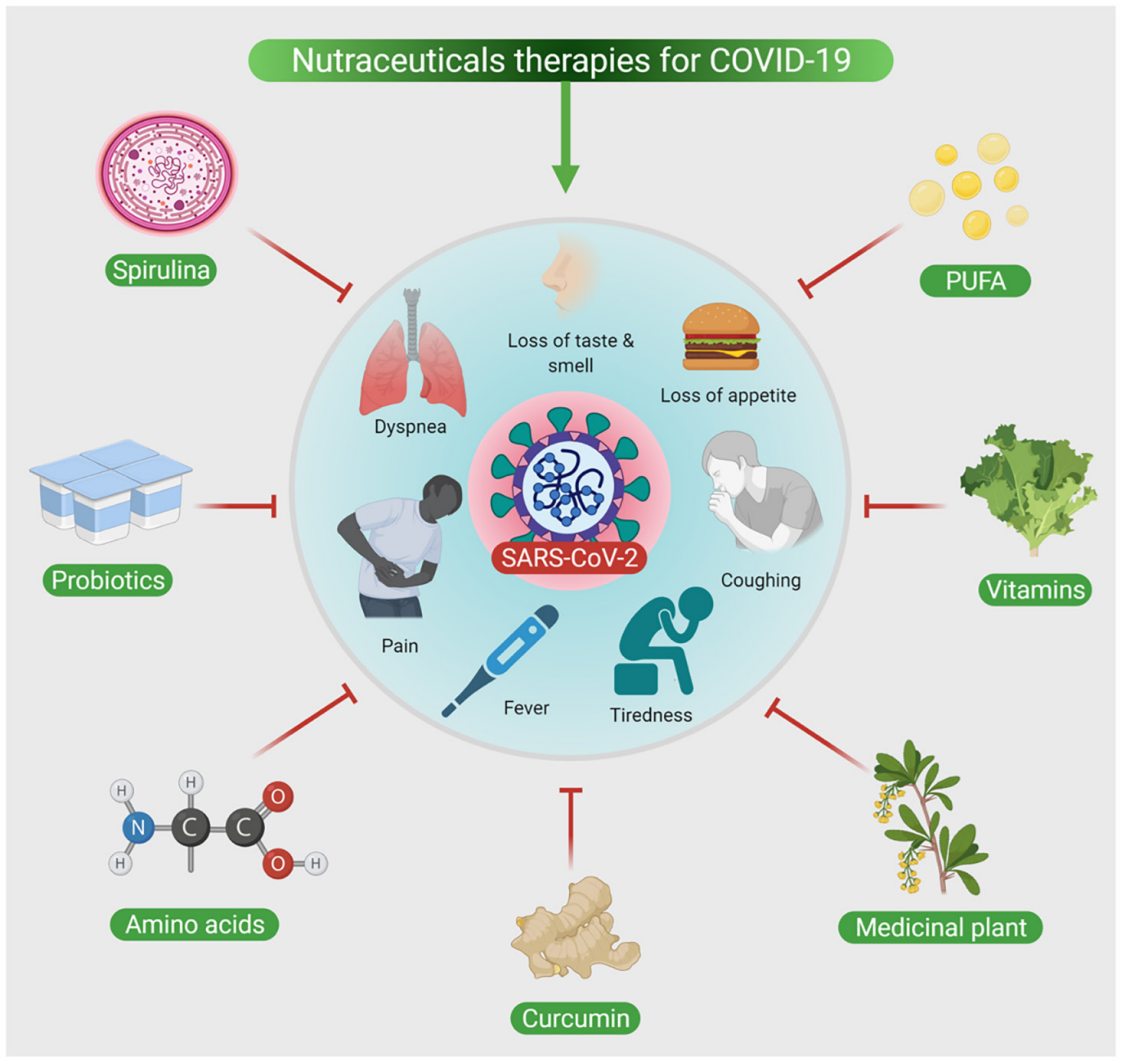
Management of COVID‐19 symptoms by nutraceuticals

COVID‐19 is associated with a condition “cytokine storm” induced by the virus that results in a massive release of proinflammatory cytokines leading to severe inflammatory symptoms in various organs including the lungs (Ragab et al., 2020). Apart from this, COVID‐19 infection also poses a high risk of patients being malnourished. Therefore, an effective nutritional approach could be one of the promising strategies to alleviate the worsening of various acute symptoms. There are reportedly various nutraceutical therapies implemented in hospital settings to improve patients' health. The body's immune response to rapidly replicate the corona viral proteins leads to symptoms of fever, pain, malaise, dyspnoea, loss of appetite, and dry cough (Donma & Donma, 2020; Parasher, 2021). In some cases, nausea/vomiting and diarrhea could precede fever and respiratory symptoms (Derosa et al., 2021). Several nutraceuticals including probiotics, polyphenols, high molecular weight polysaccharides, lectins, and berberine are being proposed to be protective agents against the symptoms of COVID‐19 in infected patients. Some of these nutraceuticals are reported to be promising prophylactic agents and they could possibly reduce the burden and severity of the COVID‐19 disease (Derosa et al., 2021). Commercially available nutraceutical products such as selenium, zinc, ascorbic acid/vitamin C, and vitamin A have possibly benefited from reducing acute symptoms by boosting the immune response (Jayawardena et al., 2020). There are plenty of herbal plant‐based nutraceutical formulations that could be potentially used to target each of the symptoms of COVID‐19. A recent review has proposed several Traditional Chinese nutraceutical‐based approaches for controlling fever symptoms in COVID‐19 patients by including medicinal plants such as *Bupleuri radix*, *Scutellariae radix*, *Chuanxiong rhizome*, *Cinnamomi ramulus*, *Forsythiae fructus*, and *Lonicera japonica* that possess significant antipyretic activity. Furthermore, single compounds such as Saikosaponin A, Baicalin, Baicalein, Ephedrine, and Geniposide are also well known for their antipyretic activity (Ma et al., 2021). Some of these herbal‐based medicines are combined to formulate a complex prescription (for example, Qingkailing injection and Shuang‐Huang‐Lian Preparation) for the treatment of fever (Gao et al., 2013, 2014). Similarly, for the management of pain, plants such as *Nigella sativa* could be beneficial as this plant is already well known to possess analgesic and antipyretic properties (Oskouei et al., 2018). α‐Hederin and nigellidine are the active ingredients of *Nigella sativa* and multiple in silico studies have proven that these compounds could be possible inhibitors of SARS CoV‐2 (Koshak & Koshak, 2020). A clinical trial involving *Nigella sativa* supplement to investigate its efficacy in mild symptomatic COVID‐19 patients is ongoing (Koshak et al., 2020). A recent systematic review also highlighted those Chinese herbal medicines can be used in the treatment of mild to moderate COVID‐19 and they are very effective in the reduction of symptoms of fever and cough in COVID patients (Du et al., 2021). The illustration (Figure 1) shows the role of Nutraceuticals in the management of COVID‐19.

Loss of appetite during COVID is due to the leptin hormone and garlic is known to recover the appetite by decreasing the leptin concentrations in the body (Donma & Donma, 2020). A naturopathic treatment strategy consisting of a multi‐nutrient, herbal and probiotic‐based treatment was designed to investigate its efficacy in mild and moderate COVID‐19 patients. The primary aim of this approach was to measure the safety of the treatment while the secondary aim was to observe an alteration in symptoms, progression to disease severity, the incidence of long COVID, and time taken for recovery. Interestingly, this treatment approach appeared to be well‐tolerated without any side effects and there was a resolution of symptoms associated with COVID‐19 infection in all patients suggesting the clinical benefit of this strategy and warrants further clinical trials with COVID‐19 cases (Barber et al., 2021). Nutraceuticals are also proven to be beneficial to non‐intensive care unit (ICU) COVID patients with loss of appetite. A recent study from Italy during the COVD‐19 pandemic investigated a short age‐adjusted nutritional risk screening in 94 non‐ICU patients (68 of these were >70 years of age). These patients received a personalized nutritional plan based on their body conditions and the plan consisted of a high‐protein and high‐calorie pureed diet, oral nutritional supplements, and/or artificial nutrition. The nutritional treatment was well tolerated by the patients. Unfortunately, 19.1% of the patients were dead and they were mostly elderly women with high body mass indices. Seventy‐two patients scored positive on at least one nutritional risk screening item. Notably, the patients whose overall energy and protein needs were not met were elderly and they had high mortality than patients whose energy needs were fulfilled. This clinical experience of a nutritional plan for COVID‐19 highlights the importance of nutraceuticals for patients with COVID‐19 (Formisano et al., 2021). Collectively, this information suggests that nutraceuticals can be a promising approach to the treatment of acute symptoms of COVID.

### 3.2 Nutraceuticals and immune response—innate, adaptive, and combined

#### 3.2.1 Probiotics

Probiotics are already well established as beneficial nutraceuticals to the host to battle against a range of diseases as they help to modulate local immunity (by maintaining the well‐being of the gut microbiome and boosting the gut health) and systemic immunity (by stimulating specific/non‐specific immune response). Therefore, the use of probiotics is practical to prevent or combat several illnesses including virus infections (Kanauchi et al., 2018). According to the Food and Agriculture Association, probiotic bacteria must have the following features; (a) the ability to survive and grow in the intestine; (b) impart favorable activity in the body; (c) non‐toxic and non‐pathogenic; (d) protect from microorganisms that are known to cause harm through various mechanisms; (e) lack transferable antibiotic resistance (Imran et al., 2022; Lopez‐Santamarina et al., 2021; Morelli & Capurso, 2012). Probiotics can regulate both innate and adaptive immune systems by modulating the functions of key immune cells such as dendritic cells, macrophages, and T and B lymphocytes (Gasmi et al., 2021). Probiotics primarily exert two different immunomodulatory functions: first, their immunostimulatory function which involves interleukin (IL)‐12 production [a cytokine that regulates the T‐cell and natural killer (NK)‐cell responses, stimulates interferon‐γ (IFN‐γ) production, and helps T helper 1 (Th1) differentiation] and second, their immunoregulatory effect that includes IL‐10 stimulation and Treg cell activation by Th2, dendritic cells, B cells, and monocytes for adaptive immunity of the host (Chiba et al., 2010; Trinchieri, 2003). The combination therapy consisting of probiotic strains of *Lactobacillus* and *Bifidobacterium*, vitamins, and minerals were found to be beneficial over control therapy (vitamins and minerals) to decrease the duration and the severity of an influenza‐induced common cold. Intriguingly, this therapy demonstrated an increase in the number of key immune cells; T‐helper cells, cytotoxic T cells, and T suppressor cells (de Vrese et al., 2006). During the asymptomatic stage and acute symptoms stage of COVID‐19 infection, boosting immune responses by probiotics to eliminate the virus and halt disease progression to severe stages is extremely beneficial through a network of signals mediated by probiotics of the bacterial origin or their cell wall structure (Sundararaman et al., 2020). Taken together, we can speculate that probiotics are a good option for the therapeutic management of COVID and long‐COVID. However, the current evidence is too premature to make any valid conclusions on the applications of a specific probiotic strain.

#### 3.2.2 Vitamin D

Vitamin D is another well‐known nutraceutical candidate that stimulates innate immunity and modulates acquired immunity in diseases such as tuberculosis and its supplement can help in the prevention of acute respiratory tract infections (Brighenti et al., 2018; Prietl et al., 2013). Epithelial cells of the airway constitutively express the vitamin D receptor that enables the protective function of vitamin D against pathogens. Vitamin D may target cell signaling pathways involved in airway inflammation to show beneficial activity in respiratory diseases such as asthma and lung infection (Agrawal et al., 2013). An in vitro experiment has revealed that vitamin D inhibits the nuclear factor kappa B (NF‐κB) pathway activation through the up‐regulation of NF‐κB inhibitory protein IκB kinase β (IKKβ) (Chen et al., 2013). The inhibition of the NF‐κB pathway by Vitamin D is also confirmed in a mice model of allergic asthma induced by the allergen ovalbumin. Vitamin D also reduces the levels of pro‐inflammatory type 1 cytokines including IL‐12, IL‐16, IL‐8, tumor necrosis factor (TNF)‐α, IFN‐γ and increases type 2 cytokines including IL‐4, IL‐5, IL‐10, and regulatory T cells (Jeffery et al., 2009; Lemire et al., 1995). As mentioned earlier COVID‐19 causes cytokine storm. Vitamin D could be another promising nutraceutical supplement for regulating the worsening of symptoms by inhibiting these pro‐inflammatory cytokines. It has been observed that human bronchial epithelial cells infected by the SARS‐CoV virus generate various NF‐κB‐mediated cytokines, including IL‐6 and IL‐8. Therefore, Vitamin D could be a promising nutraceutical to mitigate airway inflammation (Yoshikawa et al., 2009). Apart from controlling inflammation, Vitamin D also upregulates the levels of antioxidant gene nuclear factor, erythroid 2‐related factor 2 (*Nrf2*) to prevent mitochondrial stress, lipid peroxidation, protein oxidation, and DNA damage (Wimalawansa, 2019). As deficiency of vitamin D is common, to reduce the risk of infections such as influenza and COVID‐19, it is recommended to take 10,000 IU/day of vitamin D_3_ for a few weeks to increase the level of 25 dihydroxy vitamin D [25(OH)D] in the body, followed by tapering down the dose to 5000 IU/d until the goal to increase 25(OH)D level to more than 40–60 ng/ml (100–150 nmol/L) is achieved (Grant et al., 2020). This high‐dose vitamin D is reported to be effective, tolerable in high‐risk older patients, and immediately accessible for the treatment of COVID‐19 (Annweiler et al., 2020). A pilot study has shown that a high dose of calcifediol or 25(OH)D administered to COVID‐19 patients remarkably reduced the need for ICU treatment during hospitalization. While 25(OH)D seems to decrease the disease severity, larger cohort studies with groups properly matched will be required to validate the use of 25(OH)D in COVID‐19 (Entrenas Castillo et al., 2020). Nevertheless, the literature acknowledges that specific high‐quality data are needed to demonstrate the efficacy of vitamin D supplementation in the prevention of COVID‐19 (Zemb et al., 2020).

#### 3.2.3 Vitamin C

As there are limited proven drugs for the effective management of COVID‐19, vitamin C can be an adjunctive therapy for the prevention and amelioration of COVID‐19 infection as it possesses antioxidant, anti‐inflammatory, and immunomodulating activity (Holford et al., 2020). Vitamin C has a wide range of functions, and it acts on various cells of the immune system. For example, Vitamin C inhibits various forms of T‐cell apoptosis (Campbell et al., 1999) and drastically decreases the levels of high‐sensitivity C‐reactive protein, IL‐6 (Ellulu et al., 2015). Vitamin C can boost the immune system by supporting the cells of the innate/adaptive immune system. Vitamin C is also known to accumulate in neutrophils and enhance chemotaxis, phagocytosis, and production of ROS to kill the pathogens. Vitamin C supplementation is known to prevent respiratory tract infections although there is conflicting evidence if Vitamin C prevents common cold (Carr & Maggini, 2017). A recent clinical trial has revealed that patients with COVID‐19 have fivefold less vitamin C in their blood compared to healthy volunteers (2.00 mg/L in COVID‐19 patients versus 9.23mg/L in healthy volunteers). Administration of high‐dose intravenous Vitamin C (100 mg/kg/day) to these COVID‐19 patients increased the plasma Vitamin C to 13.46mg/L (slightly higher than healthy volunteers). Ultra‐high‐performance liquid chromatography‐tandem mass spectrometry may assist to quantify Vitamin C levels in COVID‐19 patients and could be helpful to precisely measure the levels of Vitamin C and thereby calculate the dosage of Vitamin C supplementation required to bring its level to baseline (Xing et al., 2021). Another clinical trial found that low dose of Vitamin C (1000 mg once daily for a median duration of 11 days) as an adjunctive therapy in COVID‐19 patients did not benefit with respect to the mortality rate, but it lowered the incidence of thrombosis (Al Sulaiman et al., 2021). The combination therapy of Vitamin C (500mg four times daily) along with vitamin A (25,000 IU daily), B complex (one amp daily), and vitamin D (60,000 IU daily) for 7 days in COVID‐19 patients significantly decreased disease severity and inflammation. Among inflammatory cytokines, TNF‐α, IL‐6, and C‐reactive protein were significantly decreased in COVID‐19 patients through this multivitamin supplementation approach (Beigmohammadi et al., 2021). Other similar trials investigating the efficacy of high‐dose vitamin C for the treatment of severe COVID‐19 are ongoing and the findings will be disseminated soon (Liu et al., 2020).

#### 3.2.4 Zinc (Zn)

Zinc has diverse biological functions in our body and is therefore considered as an essential substance. It is involved in various cellular processes such as growth, DNA synthesis, RNA transcription, as a cofactor, and as a structural element (Read et al., 2019). Zinc possesses immune‐boosting activity and exerts antiviral and antioxidant functions (Read et al., 2019). Due to these properties, it has the potential to be a supportive treatment in COVID‐19 patients (Shakoor et al., 2021). It has been proposed that zinc could enhance the efficacy of other treatment modalities such as hydroxychloroquine which is currently under investigation (Rahman & Idid, 2021). Experimental animal models have shown that Zn deficiency leads to oxidative stress, production of TNF‐α, increased expression of vascular cell adhesion molecule and results in airway remodeling that can be partly reversed by the supplementation of Zinc (Biaggio et al., 2010). Similarly, in vitro studies have shown that Zinc deficiency results in increased IL‐6, IL‐1β production, and increased expression of intercellular adhesion molecule 1 that helps in leukocytes' extravasation (Wong et al., 2015). A recent cross‐sectional study reported that serum samples from 25 out of 34 (73.5%) COVID‐19 non‐survivors and 40.9% (56 out of 137) of survivors had their Zinc levels below the threshold for Zinc deficiency (<638.7 μg/L) (Heller et al., 2021). Therefore, Zinc supplementation may be helpful to prevent “cytokine storm” in COVID‐19. Moreover, Zinc enhances IFN‐α function by an order of magnitude that can be helpful to battle IFN antagonism by SARS‐CoV‐2 proteins (Berg et al., 2001). In COVID‐19 patients, excessive amounts of circulating neutrophils (neutrophilia) is observed (Qin et al., 2020). It is known that Zinc gluconate supplementation can reduce airway neutrophil infiltration (Morgan et al., 2011). The level of Zinc in the body is a critical factor that determines anti‐viral immunity, as Zinc deficient subjects are considered at a risk of acquiring viral infections (for example: hepatitis C and HIV) (Read et al., 2019). Zinc is also known to influence virus entry into the cells, fusion, rapid replication, viral protein translation, and virus budding of respiratory viruses (Lazarczyk & Favre, 2008; Read et al., 2019) An in vitro study has shown that Zn^2+^ can inhibit coronavirus/SARS‐CoV and arterivirus RNA polymerase activity and can impair the viral replication (te Velthuis et al., 2010). A pilot study (phase IIa trial) investigating the safety and feasibility of high‐dose intravenous zinc (HDIVZn) in COVID‐19 patients admitted to the hospital found that administration of HDIVZn (*n* = 15) and placebo (*n* = 18) per day for 1 week to 15 COVID‐19 patients increased the Zinc levels above the deficiency threshold of 10.7 μmol/L (*p* < .001) on Day 6 in the HDIVZn group but not in the placebo group. HDIVZn administration to COVID‐19 patients was safe, feasible, and was not associated with any adverse event as only 3 patients experienced minimal infusion site irritation (Patel et al., 2021). Thus, Zinc could be considered as a promising supportive therapy against COVID‐19 disease due to its immunomodulatory and antiviral activities (Zhang & Liu, 2020).

#### 3.2.5 Curcumin

Curcumin has diverse beneficial functions in our physiological system such as, antioxidant, anticancer, anti‐inflammatory, and antimicrobial (bacterial, viral, fungal) (Hardwick et al., 2021; Patel et al., 2020). The antiviral activity of curcumin resulting in the inhibition of viral entry to the cell, inhibition of encapsulation of the virus, and viral protease makes it a potential candidate drug for the management of SARS‐CoV‐2 (Zahedipour et al., 2020). A recent randomized control trial evaluated the efficacy of the nano‐micellar form of curcumin formulation to modulate the inflammatory cytokines in COVID‐19 patients. Twenty COVID‐19 patients receiving 160mg of nano‐curcumin daily for 14 days showed a significant decrease (compared to the COVID‐19 patients receiving placebo) in mRNA expression of IL‐6 and IL‐1β in activated peripheral blood mononuclear cells PBMC. Similarly, a significant decrease in IL‐6 and IL‐1β secretions (measured by ELISA) was also observed in the serum of COVID‐19 subjects receiving nano‐curcumin compared to placebo. This suggests the anti‐inflammatory role of curcumin to improve clinical outcomes and overall recovery (Valizadeh et al., 2020). Another randomized control trial found that curcumin nanomicelles administration to COVID‐19 patients decreased the serum level of IFN‐γ and IL‐17 while increasing the levels of IL‐4 and TGF‐β compared to the placebo group. This suggests that nano‐curcumin formulation can be administered to COVID‐19 patients in the inflammatory phase to hasten the recovery (Hassaniazad et al., 2021). As powder curcumin form has very low bioavailability, researchers/pharmaceutical industries are designing various highly bioavailable forms of curcumin to increase cellular uptake and efficacy. CurcuRouge^TM^ is a recently developed curcumin formulation with enhanced bioavailability. A double‐blind randomized control trial found that administration of CurcuRouge^TM^ for 4 weeks significantly decreased the elevated neutrophil/lymphocyte levels in healthy elderly subjects (Kishimoto et al., 2021). As COVID‐19 patients have elevated neutrophil/lymphocyte ratio, CurcuRouge^TM^ could be a better alternative to treat COVID‐19 patients (Zeng et al., 2021). Collectively, these clinical studies found potent immune‐boosting and anti‐inflammatory effects of curcumin. Therefore, curcumin could be a beneficial nutraceutical supplement to combat inflammatory diseases due to “cytokine storm” during a COVID‐19 infection (Table 1).

**TABLE 1:** Nutraceuticals targeting immune response—innate, adaptive, and combined

Nutraceuticals	Study model	Findings	Reference
Probiotics	Probiotics strain of *Lactobacillus* and *Bifidobacterium*, vitamins, and minerals	Decrease the duration and the severity of influenza‐induced common cold. This therapy was able to increase the number of key immune cells; T‐helper cells, cytotoxic T cells, and T suppressor cells	(de Vrese et al., 2006)
	Probiotics strain of *Lactobacillus plantarum* NCIMB 8826 against RSV, pneumovirus	TLR‐dependent inflammatory response	(Al Kassaa et al., 2014)
	Probiotic strain of *Lactobacillus rhamnosus* M21 against pneumonia and influenza	Increases interferon‐γ and interleukin‐2	(Song et al., 2016)
	Probiotic strain of *Lactobacillus casei* DN‐114001aganist RTI, rhinopharyngitis, and influenza	Enhanced defensin expression and innate immunity	(Guillemard et al., 2010)
Vitamin	Patients aged ≥ 65 years with COVID‐19 (high‐risk older patients)	High dose of calcifediol or 25(OH)D administered to COVID‐19 patients remarkably reduced the need for ICU treatment during hospitalization	(Annweiler et al., 2020)
	To evaluate the efficacy and safety of using ascorbic acid in supplemental doses as adjunctive therapy for critically ill (ICU admitted) COVID‐19 patients	Low dose of Vitamin C (1000 mg once daily for a median duration of 11 days) as an adjunctive therapy in COVID‐19 patients did not benefit in mortality rate but it lowered the incidence of thrombosis	(Al Sulaiman et al., 2021)
Multivitamin supplement	The combination therapy of vitamin C (500mg four times daily) along with vitamin A (25,000 IU daily), B complex (one amp daily), and D (60,000 IU daily) for 7 days in COVID‐19	The combination therapy significantly decreased disease severity and inflammation. Among inflammatory cytokines, TNF‐α, IL‐6, and C‐reactive protein was significantly decreased in COVID‐19 patient by this multivitamin supplementation approach	(Beigmohammadi et al., 2021)
Zinc	In vitro study on Vero‐E6 cells infected with SARS‐CoV	Zn^2+^ inhibits coronavirus/SARS‐CoV and arterivirus RNA polymerase activity and impairs the viral replication	(te Velthuis et al., 2010)
	A pilot study (phase IIa trial) investigating the safety and feasibility of high‐dose intravenous zinc (HDIVZn) in COVID‐19 patients admitted to hospital	Administration of HDIVZn and placebo per day for 1 week to 15 COVID‐19 patients increased the level Zn level above the deficiency threshold of 10.7 μmol/L on Day 6 in the HDIVZn group but not in the placebo group. HDIVZn was found to be safe, feasible, and not associated with any adverse events as only 3 patients experienced minimal infusion site irritation	(Patel et al., 2021)
Curcumin	To identify the effects of nano‐curcumin on the modulation of inflammatory cytokines in COVID‐19 patients	COVID‐19 patients receiving 160mg of nano‐curcumin daily for 14 days showed a significant decrease (compared to the COVID‐19 patients receiving placebo) in mRNA expression of IL‐6 and IL‐1β in activated peripheral blood mononuclear cells PBMC. Similarly, a significant decrease in IL‐6 and IL‐1β secretion (measured by ELISA) was also observed in serum of COVID‐19 receiving nano‐curcumin compared to placebo	(Valizadeh et al., 2020)
Berbamine, an alkaloidal moiety presents in the plant *Berberis amurensis*	In vitro study to investigate if berbamine effectively inhibited the entry of SARS‐CoV‐2‐S or MERS‐CoV‐S into host cells (primary human lung fibroblasts and Vero‐E6 cell line)	Berbamine efficiently protected the cells from different coronaviral (SARS‐CoV‐2 and MERS‐CoV) infections by inhibiting the transient receptor potential cation channel, mucolipin subfamily (a Ca^2+^ permeable non‐selective cation channels in endosomes and lysosomes) leading to impairment in endolysosomal trafficking of viral receptors ACE2 and DPP4	(Huang et al., 2021)
Resveratrol and its analog pterostilbene	In vitro study on air‐liquid interface cultured human primary bronchial epithelial cells to test the efficacy of resveratrol and pterostilbene to inhibit SARS‐CoV‐2 replication	Resveratrol and its analog pterostilbene inhibited the SARS‐CoV‐2 viral replication in human primary broncho epithelial cells up to 48 hours post‐infection	(Ter Ellen et al., 2021)

### 3.3 Management of extra‐pulmonary complications in COVID‐19 by nutraceuticals

#### 3.3.1 Mental health—neurotransmitters, anxiety, depression

COVID‐19 systems are accompanied by short‐ and long‐term neuropsychiatric symptoms and long‐term brain sequelae. In addition, few patients also experienced loss of sense of smell, increased stress levels, cognitive and attention deficits, symptoms of anxiety, depression, seizures, and even suicidal behavior (Cheng et al., 2004; Lee et al., 2007; Meinhardt et al., 2021; Woo et al., 2020). These symptoms present prior, during, and after patients develop respiratory symptoms (Woo et al., 2020) suggesting independent brain damage. To date, few studies have evaluated the possible mental health outcomes of COVID‐19 infection demonstrating that in patients with COVID‐19 during their acute phase had a prevalence of 96.2% of post‐traumatic stress disorder symptoms (Bo et al., 2021), 34.72 and 28.47% of anxiety and depression symptoms respectively. Moreover, few studies have also reported post‐COVID‐19 neuropsychiatric symptoms in 20% to 70% of patients lasting for longer periods even after respiratory symptoms were resolved, suggesting the involvement of the brain (Woo et al., 2020). Taken together, these data indicate that infection with COVID‐19, can yield an adverse impact on the well‐being of mental health both short‐ and long‐term.

#### 3.3.2 COVID‐19‐related inflammation and neurotoxicity

Interestingly, studies have reported that patients with severe COVID‐19 infection experience increased serum levels of pro‐inflammatory cytokines including IL‐1β, IL‐6, IL‐10, and TNF‐α. This increase in cytokines could damage the BBB thus affecting its permeability that enables TNF‐α to directly cross the blood‐brain barrier (BBB) leading to the activation of microglia and astrocytes (Liddelow et al., 2017; Pan & Kastin, 2007). These activated microglia secrete inflammatory mediators, including glutamate, quinolinic acid, complement proteins, ILs, and TNF‐α (Vasek et al., 2016). Importantly, an increase in quinolinic acid results in higher glutamate levels and upregulation of NMDA receptors (glutamate and ion channel protein receptor), conceivably inducing altered memory, neuroplasticity, and hallucinations.

Moreover, increased inflammation activates key enzymes such as indoleamine dioxygenase, that ultimately, metabolizes tryptophan to kynurenine instead of serotonin (Roman & Irwin, 2020). Indeed, neurotransmitter release was affected in interferon (IFN)‐α treated patients as they presented with depression and fatigue severity. Since inflammation leads to reduced neurotransmission, cognitive and psychomotor activity, depression, and suicidal behavior, in patients, studies have shown that they poorly respond to traditional anti‐depressants (Roman & Irwin, 2020). Studies have found that individuals with major depressive disorder have elevated levels of IL‐1 and IL‐6 in blood and cerebrospinal fluid (CSF) and C‐reactive protein in serum. This suggests that elevated levels of ILs have a significant correlation with brain glutamate levels in patients (Boldrini et al., 2019; Mahajan et al., 2018). Thus, neuroinflammation may contribute to reduced activity of neurotransmitters and increased excitotoxicity (Roman & Irwin, 2020).

#### 3.3.3 Management of mental health by nutraceuticals

##### 3.3.3.1 Omega 3 denosyl‐Methionine

Epidemiological studies have reported that the prevalence of decline in anxiety and depression is associated with a high intake of ω‐3 polyunsaturated fatty acids (ω‐3 PUFAs). The major source of this substance is fish. Interestingly, countries with higher fish consumption support the finding that fish oil may be a key source to prevent mental health disorders such as anxiety and depression as they are less common in those countries.

Importantly, studies have shown that G‐proteins within the lipid rafts are the main sites of action where PUFAs at the cell membrane interact with. Given that it interacts with the PUFAs present at the cell membrane, every change in protein location at this site will be able to translate alterations in the signaling of neurotransmitters (Burhani & Rasenick, 2017). In addition, these proteins are also key targets for anti‐depressant agents. This suggests that the lipid rafts may play an important role in the treatment efficacy and therapeutic outcomes (Burhani & Rasenick, 2017; Djuricic & Calder, 2021; Torres et al., 2020). A meta‐analysis and meta‐regression study observed that higher dose supplementation of ω‐3 PUFAs particularly, eicosapentaenoic acid (EPA) in patients with mental health disorders had a significant clinical benefit (Liao et al., 2019; Mocking et al., 2016). Thus, the findings from various studies indicate that ω‐3 PUFAs with EPA may have pleiotropic effects including anti‐inflammatory action and neuroprotective effect (i.e., the modification of signaling membrane proteins and the endocannabinoid effect); all of which contribute to anti‐depressant action. Although these pleiotropic effects have been appreciated by various authors, the elucidation of these mechanisms will prove beneficial.

##### 3.3.3.2 Vitamin D

The main source of fat‐soluble Vitamin D specifically Vitamin D3 (also known as 1, 25‐dihydroxycholecalciferol or calcitriol), i.e., 7‐dehydrocholesterol is the sunlight. It is produced in the skin upon exposure to sunlight (Mostafa & Hegazy, 2015). Vitamin D3 is more efficient and is mainly found in fish oil, cod fish liver, or milk. Interestingly, clinical studies have demonstrated that vitamin D deficiency in patients with depression has an association with the symptoms and this is because vitamin D can influence the levels of serotonin and melatonin thereby, affecting mood, and sleep patterns (Gao et al., 2018; Huiberts & Smolders, 2021). Notably, studies have demonstrated the inverse relationship between vitamin D and melatonin thus indicating that both may share common mechanisms that could be relevant for brain disorders (Gao et al., 2018; Huiberts & Smolders, 2021).

Moreover, vitamin D mediates the synthesis of brain serotonin from amino acids such as tryptophan which ultimately, activates the transcription of the serotonin‐synthesizing gene tryptophan hydroxylase 2 (TPH2) in the brain. Thus, vitamin D in combination with tryptophan supplementation may prove to be a practical therapy for depression. Importantly, studies have shown that imbalance in vitamin D, EPA, or DHA levels can disturb serotonin activation and function significantly (Patrick & Ames, 2014, 2015). Nevertheless, optimal concentrations of vitamin D can enhance the synthesis of serotonin that mimics Monoamine oxidase (MAO) inhibitors thus, increasing serotonin in the central nervous system (CNS) (Sabir et al., 2018). These findings suggest that vitamin D is crucial for brain function. However, further elucidation is required in deriving the optimal time of administration, and its dosage based on inter‐individual requirements (Patrick & Ames, 2015; Sabir et al., 2018).

##### 3.3.3.3 Methylfolate

Studies have shown that patients with depression have reduced levels of folate in their serum and diet (Bender et al., 2017). Interestingly, genetic polymorphism in the Methylenetetrahydrofolate reductase (MTHFR) gene, the MTHFR C677T variant is associated with an increased risk of depression and other psychiatric disorders (Wan et al., 2018). Thus, supplementation with folate and its different forms such as 5‐MTHF has proven to be effective in the treatment of patients with depression (Fava & Mischoulon, 2009). Current studies support the possible use of 5‐MTHF in the clinical management of mental disorders, prominently as adjunctive therapy in combination with anti‐depressants (Fava & Mischoulon, 2009).

##### 3.3.3.4 Creatine and amino acids

Creatine is an organic compound naturally synthesized in our cells, also found exogenously in different food products, such as fish and meat. Creatine is a well‐known supplement among athletes, as this component exerts a plethora of ergogenic benefits. However, recent studies have elucidated the central role of creatinine which is an organic compound naturally synthesized in human cells, and also found exogenously in various foods such as fish and meat. Studies have also reported the role of this substance in health and disease conditions as it has an array of targets both at the cellular and molecular levels (Kreider & Stout, 2021). The key effects of creatine include its role in the regulation of metabolic activities by decreasing the production of reactive oxygen species (ROS) and also as a potential anti‐inflammatory agent by diminishing homocysteine levels (Clarke et al., 2020). The synthesis of creatine occurs in the liver, kidney, and pancreas from amino acids such as glycine, methionine, and arginine. Indeed, studies have shown that creatine is a central molecule that is involved in the metabolism of amino acids, notably in the met cycle, where its synthesis account for 40% (Brosnan et al., 2011). In particular, with the brain, creatine supplementation is associated with significant benefits in regulating cognitive function, especially post‐deficiency that is induced by acute stressors such as exercise, stress, sleep deprivation, or under chronic pathological conditions (Balestrino & Adriano, 2019). Studies have also shown that patients with depression have demonstrated a reduced intake of dietary creatine thus affecting their creatine levels overall (Bakian et al., 2020) and abnormal metabolism of creatine in the brain (Pazini et al., 2019). Thus, supplementation of creatine may be a potential therapeutic approach for depression. Studies have also shown that amino acids such as phenylalanine and tyrosine both are key regulators in the synthesis of neurotransmitters, dopamine, and norepinephrine (Lakhan & Vieira, 2008), thus, affecting the neuroinflammatory response in the brain of patients suffering from depression (Miller & Raison, 2016; Strasser et al., 2017). Indeed, studies have shown that patients with low levels of dopamine may benefit from the supplementation of tyrosine (Mouret et al., 1988). Notably, clinical studies have shown that higher consumption of dietary tryptophan reduces symptoms related to depression due to its direct effects on the synthesis of serotonin synthesis (Kikuchi et al., 2021; Lindseth et al., 2015; Turner et al., 2006). Thus, tryptophan may be a potential therapy for depression.

##### 3.3.3.5 Plant‐derived bioactive compounds

Growing evidence suggests that bioactive compounds such as polyphenols that are synthesized by plants act as strong antioxidants in the management of various diseases (Gonzalez, 2020; Singla et al., 2019). Polyphenols are classified into two subtypes, that is, flavonoids and non‐flavonoids. Quercetin is a key flavonoid that possesses antidepressant properties including (Panche et al., 2016) an improved functioning of the monoaminergic system, GABAergic transmission, brain‐derived neurotrophic factor (BNDF), and amelioration of the neuroinflammatory response in the brain (Hritcu et al., 2017). Notably, curcumin—a non‐flavonoid compound present in *Curcuma longa* has a beneficial effect in the treatment of depression. Apart from the anti‐oxidant and anti‐inflammatory properties, Curcumin also has shown its role in the improvement of BDNF activity, serotoninergic, and dopamine transmission (Trebaticka & Durackova, 2015). Indeed, studies have shown that Curcumin may be a potential therapy in the clinical management of anxiety and depression (Fusar‐Poli et al., 2020).

#### 3.3.4 COVID‐19 and depression/peripheral neuropathy symptoms/cognitive impairment

Globally, several cohort studies conducted in hospitals have reported an association between neuro‐behavioral implications with COVID‐19. Anosmia, ageusia, anxiety, stress, and depression are some of the neurological manifestations reported in patients suffering from COVID‐19. Some of the symptoms have a direct relation to a viral infection, and some of them are results of changes in lifestyle as well as due to certain required restrictions during lockdown that have caused quarantine blues. Quarantine blues further lead to anxiety, which is further associated with disturbance in sleep pattern, duration, and depression. Moreover, SARS‐CoV‐2 is associated with inflammatory responses causing the excessive generation and release of cytokines. This has led to the development of other comorbidities. Neuronal implications and their involvement in COVID‐19 mainly correspond to three situations, that is, (1) neurological manifestations of viral infection, (2) infection in patients with neurological co‐morbidity, and (3) post‐infective neurological complications (Sharma et al., 2021). All these factors have created a major impact on mental health apart from the pulmonary system and have triggered neuro‐immunological reactions. It is well established that anxiety‐induced inflammatory responses are not only limited to cellular immunity but also associated with humoral immunity. As a consequence, it is suggested evidently that individuals suffering from chronic anxiety are more susceptible to infections and inflammatory responses (Rammal et al., 2010). Although there is no standard line of treatment available for COVID‐19 worldwide, physicians recommend nutraceuticals in order to boost immunity and for controlling neuro‐behavioral implications (Sharma et al., 2021). Nutraceuticals have dual applications including preventive and therapeutic. During previous similar COVID‐19 outbreaks like SARS‐CoV and MERS‐CoV, few nutraceuticals were studied and observed to have a significant influence on the prevention of the occurrence of the disease (Savant et al., 2021). Since ancient times, due to their anti‐viral property and potential benefits on the nervous system, nutraceuticals are widely used for prophylaxis and as an adjunct therapy to deal with various viral infections and associated neurological manifestations (Subedi et al., 2021). It is evident that the functional network and activity of the brain are related to mood, depression, and anxiety which are mediated by neuronal signaling. The neuronal signaling is further associated with the release of neurotransmitters at synapses. Several nutraceuticals and dietary supplements have proven to be a rich source of various amino acids (tyrosine and tryptophan) which are precursors of several neurotransmitters like dopamine, norepinephrine, and serotonin. Studies and clinical trials have shown that supplementation of these agents could improve cognitive performance during stressful conditions. Several water‐soluble vitamins (e.g., vitamin C), fat‐soluble vitamins (vitamin D), minerals (zinc, magnesium, manganese, and selenium) along with plants (*Bacopa monnieri*, *Withania somnifera*, and *Curcuma longa*) that contain several bioactive substances such as alkaloids, and flavonoids are proven to be beneficial. These bioactive compounds are also observed to have potential neuroprotective benefits (Ghosh, 2021). Plant‐based nutraceuticals like apigenin and luteolin, which belong to the class of flavonoids, are suggested to activate the nitric oxide signaling pathway and regulate cyclic guanosine monophosphate levels (Roberts et al., 2013). Consequently, it causes vascular relaxation resulting in lowering the elevated blood pressure and heart rate during sleep. These are the important processes of non‐rapid eye movement (REM) sleep, thus these flavonoids and related biomolecules influence sleep patterns and anxiety processes (Nami et al., 2020). Certain plant‐based bioactive compounds like valerenic acid, mahanimbine, and coumarins have sedative, anxiolytic, and antidepressant activities (Batra et al., 2018; Jung et al., 2015; Sharma et al., 2017). Studies have shown that valerenic acid has benzodiazepine‐like tranquilizing activity which acts by increasing the levels of gamma‐aminobutyric acid (GABA). Therefore, valerenic acid produces a calming effect along with the anxiolytic activity.

Among the vitamins, vitamin D is widely studied to explain its role in brain health. Studies have explained that vitamin D reduces the increased levels of calcium in neurons which is one of the important factors of depression. Also, it is suggested by several researchers that vitamin D regulates the survival of hippocampal neurons through the release of nerve growth factors. Vitamin D insufficiency is also correlated with cognitive impairment, psychosis, and an alteration in the level of neurotransmitters (Berridge, 2017; Gezen‐Ak et al., 2014). Minerals, namely zinc have been demonstrated to have antidepressant activity. Zinc is a trace mineral required for the normal function of the brain which also includes the uptake of neurotransmitters like serotonin and acts as a cofactor to various enzymatic reactions including neurotransmitter activity in the central nervous system (Levenson, 2006). Several studies including clinical trials have reported that zinc has a protective effect against brain damage as well as improves behavioral and cognitive activities (da Silva et al., 2021; Partyka et al., 2011). Zinc levels can be applied as a biomarker to determine depression or anxiety, as reduced levels of zinc have been observed in depressed patients. Possible hypotheses suggested in several studies for this correlation are (1) anxiety‐induced activation of immunity and elevated inflammatory responses lead to utilization of zinc that consequently causes a reduction in the level of zinc. (2) Increased production of cortisol in depressed patients promotes the synthesis of metallothionein (a zinc‐binding protein) causing the reduction in zinc levels (Levenson, 2006).

##### Turmeric extracts

Studies have shown that turmeric greatly improve musculoskeletal pain with less side effects as well as adverse effects compared to NSAIDs such as Ibuprofen (Chandran & Goel, 2012; Daily et al., 2016; Kuptniratsaikul et al., 2009, 2014). Furthermore, propriety formulations containing turmeric extracts have been developed across the globe by drug manufacturers that have proven to impart a promising effect on improving musculoskeletal health. In addition, several studies have demonstrated that curcumin (BCM‐95®) itself or in combination with NSAIDs such as diclofenac sodium showed greater disease activity scores, improved inflammatory biomarker levels such as C‐reactive protein (CRP) and erythrocyte sedimentation rate (ESR), compared to diclofenac sodium alone (Chandran & Goel, 2012; Shep et al., 2019). Notably, studies have also shown that herbal formulations containing substances like curcumin, gingerols, and piperine, can aid in reducing prostaglandin E2 levels in patients that have musculoskeletal pain to the same extent as NSAIDs (Heidari‐Beni et al., 2020). There is growing evidence of turmeric extracts showing anti‐inflammatory properties such as, decreased production of pro‐inflammatory cytokines such as TNF‐α, IL‐1β, IL‐8, IL‐6, and structural degradation proteases such as matrix metalloproteinases (MMPs), collagenase, and anti‐oxidant properties (Bengmark, 2006; Heidari‐Beni et al., 2020; Khanna et al., 2007; Kim et al., 2012; Oyagbemi et al., 2009; Saja et al., 2007; Yang et al., 2013). Turmeric extracts are well documented to inhibit the NFκB pathway and other proinflammatory signaling pathways including cyclooxygenase (COX)‐2, AP‐1, Egr‐1, STAT (signal transducers and activators of transcription), and mitogen‐activated protein (MAP) kinases (Goel et al., 2001, 2008; Reuter et al., 2011). These data undoubtedly point toward the definite effectiveness of turmeric extracts as a promising therapy in musculoskeletal pain, if administered at an early stage.

##### Terrestrial botanicals

Interestingly, terrestrial botanicals such as avocado or soybean extracts are being appreciated in literature to have shown positive therapeutic benefits in relieving musculoskeletal pain (Appelboom et al., 2001; Gluszko & Stasiek, 2016). Specifically, avocado or soybean extracts have shown to inhibit the production of cytokines such as IL‐1, IL‐6, IL‐8, and PGE‐2. Moreover, they also promote the expression of TGF‐β and activation of collagen synthesis thereby reducing the production of stromelysin (Boumediene et al., 1999; Henrotin et al., 2003). These data suggest that avocado/soybean can impact both inflammatory and structural proteins that shape the physiology of the musculoskeletal system. Similar effects have been found with the species belonging to the Ginger root family as they aid in blocking the development of inflammatory mediators such as thromboxane, leukotrienes, and prostaglandins and inhibit COX and lipoxygenase which are key factors in arachidonic acid metabolism (Lantz et al., 2007; Niempoog et al., 2012; Nurtjahja‐Tjendraputra et al., 2003; Paramdeep, 2013).

#### 3.3.5 Insomnia/sleep disorders

Treatment options for sleep disorders are mainly associated only with the management of dependence symptoms. This has led to a search for better therapeutic options such as nutraceuticals. Interestingly, various studies have implicated positive effects from nutraceuticals such as caffeine, chamomile, kava kava, cherries, and its extract, valerian, and Lemon balm in the treatment of sleep disorders as most of them are not associated with dependence or withdrawal symptoms (Andrews et al., 2007; Beaumont et al., 2004; Donath et al., 2000; Esteban et al., 2004; Hadley & Petry, 2003; Nicholson et al., 2004; Rowe et al., 2011; Rycroft, 2003).

#### 3.3.6 Cardiovascular disorders (CVD)

##### Phytosterols

Phytosterols are found in a range of plant products including fruits, vegetables, cereals, seeds, and nuts. They have a molecular skeleton similar to cholesterol thus, possessing their biological activity in treating CVD (Moreau et al., 2002).

##### Polyphenols

Polyphenols are phytochemicals such as flavonoids, phenolic acids, stilbenes, and lignans that are structurally diverse with widespread distribution in foods of plant origin (Pandey & Rizvi, 2009). Polyphenols extracted from grapes and its derivatives, tea, and cocoa are implicated in the prevention of CVD. Grapes contain phenolic compounds including anthocyanins, flavonols, stilbenes, and phenolic acids, where resveratrol (3,5,4′‐trihydroxy‐trans‐stilbene) is the most extensively studied bioactive compound (Burns et al., 2002; Spacil et al., 2008). Cocoa and its derivatives such as Theobroma cacao are widely used polyphenols in CVD (Andres‐Lacueva et al., 2008). In addition, other derivatives also include anthocyanins, catechins, flavonol glycosides, and procyanidins (Hammerstone et al., 1999; Lee et al., 2003). Polyphenols found in tea include catechins, theaflavins, tannins, and flavonoids. As per the literature, the minimally fermented green tea contains more catechins such as epigallocatechin gallate, epicatechin‐3‐gallate, epigallocatechin, and epicatechin (Khan & Mukhtar, 2007) compared to black tea that is rich in flavins and thearubigins (Balentine et al., 1997).

#### 3.3.7 Musculoskeletal disorders and the influence of nutraceuticals

Prevalence of muscle fatigue, muscle weakness, back pain, myalgia, and arthralgia are the common manifestations observed in COVID‐19. Some of the reports have also mentioned about the association of Guillain‐Barré syndrome with COVID‐19 (Caress et al., 2020). Moreover, prolonged administration of corticosteroids has also impacted bone mineral density resulting in osteoporosis and osteonecrosis (Vaishya et al., 2021). One of the possible reasons for such manifestation is the activation of aggressive inflammatory responses influenced by the viral infection. Thus, identifying the musculoskeletal manifestations associated with COVID‐19 and an early diagnosis would be beneficial to manage the condition at its earliest and this also may ensure immediate recovery. Potassium, phosphorous, zinc, calcium, vitamin D, vitamin C, vitamin B1, B2, B3, B5, and B12, vitamin K2, leucine, omega‐3 fatty acids, phosphatidic acid, folic acid, and creatine are some of the examples of nutraceuticals and dietary supplements that are reported to improve the musculoskeletal health and cognitive functions (Boros, 2013; Deane et al., 2017; Iolascon et al., 2017). All these nutraceuticals and dietary supplements are responsible to prevent osteoarthritis and autoimmune disorders and promote bone formation, regulation of proteins in bone tissue, modulating muscle contraction, collagen synthesis, initiating cell division, and osteoblast activity (Boros, 2013). Apart from all these evidence published in literature regarding the influence of nutraceuticals in managing musculoskeletal manifestations, it is necessary to have accurate information and education regarding the use of appropriate nutraceuticals. Because these are not evaluated for their safety and specific effectiveness by regulatory agencies and a proper regimen and dose intake are of utmost importance to manage musculoskeletal manifestations in an efficient way.

#### 3.3.8 Dermatological infections associated with COVID‐19

Many studies have reported where COVID‐19 infection has caused various dermatological symptoms such as rashes, itching, urticaria and other dermatitis‐related infections that affect the body, hands, and faces (Darlenski & Tsankov, 2020). In one of the studies, Yan and co‐workers reported the problems related to the skin caused by the SARS‐CoV‐2 virus. Health care personnel who have been assigned for controlling and reducing the SARS‐CoV‐2 virus infection, have the greatest risk of developing skin issues and mucous membrane damage, which may result in acute or chronic dermatitis infections, secondary infection, and aggravation of pre‐existing skin illnesses. Latex gloves are mostly employed in workplaces to prevent the spread of the SARS‐CoV‐2 virus, which causes skin softness, maceration, whitening, and wrinkles (Yan et al., 2020). During the COVID‐19 pandemic, it has been observed that lifestyle modifications such as wearing masks and hand gloves have caused several skin allergies (Yan et al., 2020). Gheisari et al., have investigated the dermatological infections caused using N95 masks. According to this study, 35.5% of medical personnel used N95 masks on a daily basis, resulting in acne, facial dermatitis, and pigmentation of the chin, cheeks, and nasal bridge. Additionally, it was found that dermatitis with pruritic lesions mostly resulted in irritation and allergic contact dermatitis that generally developed as a result of the adhesives or other components of the breathing mask, such as rubber straps and metal clips (Gheisari et al., 2020). In another study, Donovan et al., 2007 reported that N95 masks caused reactions during the time of the SARS epidemic in Toronto (Donovan et al., 2007). Urticarial facial eruptions were reported in 3 patients, dermatitis in 5 patients, and acute respiratory symptoms without skin lesions in 2 patients were observed (Sharma & Malviya, 2020).

Many other related symptoms were also observed among COVID‐19 patients during the time of the COVID‐19 pandemic such as patchy rash, blisters that look like chickenpox, a lace‐like pattern on the skin, large patch with several smaller ones, itchy bumps, round, pinpoint spots on the skin, flat spots, and raised bumps that join together (Genovese et al., 2021). Among these infections, urticaria along with angioedema i caused by several bacteria and viruses, such as herpes virus, cytomegalo virus, epstein‐Barr virus, and mycoplasma (Kanani et al., 2011). In one of the studies, Galván Casas et al., 2020 reported that urticarial rash occurred in 19% of their cohort under observation. These rashes appeared simultaneously with systemic symptoms and lasted approximately 1 week. It was observed that these were associated with medium and high severity of COVID‐19. In urticarial rash, inflammatory responses were elevated which enhanced the immune hypersensitivity response, cytokine release syndrome, SARS‐Cov2‐RNA, deposition of microthrombi as well as vasculitis along with superficial perivascular dermatitis of microthrombi (Galván Casas et al., 2020).

##### Nutraceuticals and dermatological infection associated with COVID‐19

Several nutraceuticals have been reported that may be beneficial in reducing the symptoms of dermatological infections. Among them, some of the nutraceuticals also reduce symptoms of COVID‐19 and COVID‐19‐induced dermatological symptoms. Some of the important nutraceuticals are probiotics, prebiotics (Olaimat et al., 2020), resveratrol (Liao et al., 2021), hesperidin, quercetin (Saeedi‐Boroujeni & Mahmoudian‐Sani, 2021), lactoferrin (Chang et al., 2020), vitamin A, C, D, K (Goddek, 2020; Slominski et al., 2020), Zinc (Mossink, 2020), and omega‐C fatty acid (Gutiérrez et al., 2019; Hathaway et al., 2020). The nutraceuticals discussed above are reported to attenuate the progression of inflammation, mitochondrial dysfunction, endoplasmic reticulum stress, oxidative stress, and apoptosis pathways associated with urticaria, psoriasis, rash, and other dermatological infections induced by COVID‐19. Furthermore, they also helped in the reduction of skin infections (Lordan et al., 2021; Nwanodi, 2018).

##### Resveratrol

Resveratrol promotes the nuclear factor erythroid 2–related factor 2/antioxidant responsive element (Nrf2/ARE) pathway, which mediates anti‐inflammatory, anti‐oxidative, and anti‐apoptotic effects. The cis‐acting component ARE is responsible for the transcriptional activation of protective genes against SARS‐CoV‐2 infection. Nrf2 attaches to ARE and triggers the Nrf2/ARE pathway, which protects cells against oxidative stress. Resveratrol facilitates the expression of HO‐1 via activating the Nrf2/ARE pathway, thereby attenuating the effects of NF‐κB on antioxidants and its activation of inflammatory flares. Moreover, activation of the Nrf2/ARE pathway increases the expression of SIRT1/AMPK, which results in decreased inflammation. These inflammatory mediators and oxidants are activated by SARS‐CoV‐2 viruses and cause various dermatological infections. Resveratrol protects the cells from apoptosis and reduces dermatological symptoms by attenuating these inflammatory mediators thereby producing antioxidative effects (Imran, Iqbal et al., 2020; Liao et al., 2021).

##### Vitamin D

Vitamin D and its activated hydroxyl form possess anti‐inflammatory properties (Jan et al., 2019). They induce antioxidative responses and stimulate the innate defense against infectious pathogens. Calcitriol and the non‐calcemic hydroxy derivatives produced by CYP11A1 have been reported to demonstrate these features. They suppress the production of pro‐inflammatory cytokines, downregulate NF‐κB, exhibit antagonism on ROR, and protect against oxidative stress via NRF‐2 activation. As a result, direct distribution of vitamin D hydroxy derivatives should be considered for the treatment of COVID‐19 or acute respiratory distress syndromes of various aetiologies. Additionally, COVID‐19 patients should receive a high dose of vitamin D because patients who are most susceptible to this illness are usually vitamin D deficient. It has been predicted that different modes of administration (oral and parenteral) of vitamin D or vitamin D derivatives would have varying effects on the clinical and therapeutic outcomes (Slominski et al., 2020).

##### Omega‐3 fatty acid

Omega‐3 fatty acids are a group of polyunsaturated fatty acids that stimulate immune cell activation, especially in T‐cells, B‐cells, dendritic cells, macrophages, neutrophils, basophils, natural killer cells, mast cells, and eosinophils. Omega‐3 fatty acids are critical components of the host cellular membrane. They regulate membrane fluidity and the intricate lipid raft assembly process (Hathaway et al., 2020). Omega‐3 fatty acids provide an anti‐inflammatory impact by activating the TLR4 and G‐protein coupled receptor‐120 pathways, therefore reduce the level of NF‐κB expression (Gutiérrez et al., 2019; Hathaway et al., 2020).

##### Probiotics

The administration of probiotics in COVID‐19 patients has shown to increase their immunity and general health. Probiotics such as *Lactobacillus casei* also interact with TLRs on epithelial cells, increase the generation of cytokines that are critical for enhancing epithelial cell productivity and preventing apoptosis. Hence, they promote their survival and proliferation during the restoration process (Baud et al., 2020).

## 4 CONCLUSION AND FUTURE PERSPECTIVES

Based on the current evidence from the literature, it could be concluded that there are several promising therapeutic benefits of effectively using nutraceutical‐based products in the management of viral infections such as COVID‐19. Moreover, they are affordable, possess a long shelf‐life, easy to handle and store, easy to be administered compared to other complex injectable dosage forms such as vaccines, and intravenous injections containing allopathic medicines. In order to recommend the use of these compounds as an antiviral therapy, more detailed studies are required. Existing evidence is not translatable to humans because of several important limitations. Most of the tests are performed on in vitro or cell‐free/cell‐based models, which do not necessarily produce a human response. Inadequate pharmacokinetic and toxicological studies along with not standardized or inadequately titrated extracts may also contribute to this problem. Due to differences in bioavailability and bioactivity between individuals and their different biologic responses, estimating their bioavailability and bioactivity can also be challenging. Thus, advanced in vitro and animal studies are still needed to determine the mechanisms of action, pharmacokinetics, and safety profile of the isolated bioactive compounds and plant complexes before conducting human clinical trials. Over the past two years, drug manufacturers around the world have come up with newer and more effective nutraceutical‐based formulations for infectious diseases, like COVID‐19 in particular. Patanjali, a nutraceutical‐based drug company in India has designed a commercial oral tablet dosage form named “*Coronil Kit®*” which contains various therapeutic nutraceuticals. The formulation has been claimed to boost the immune system to fight against various common viral infections. However, more in‐depth research studies are necessary to validate their therapeutic potential before they can be translated for clinical usage. These compounds can be preliminarily assessed using observational studies focusing on outcomes such as hospitalizations and mortality rates. Ultimately, support from the national health systems would be crucial to ensuring adequate provision of these nutraceuticals.

## AUTHOR CONTRIBUTIONS

**Keshav Raj Paudel**: Writing—original draft; Writing—review & editing. **Vyoma Patel**: Writing—original draft; Writing—review & editing. **Sukriti Vishwas**: Writing—original draft; Writing—review & editing. **Saurabh Gupta**: Writing—review & editing. **Sumit Sharma**: Writing—review & editing; Writing—original draft. **Yinghan Chan**: Writing—original draft. **Niraj Kumar Jha**: Writing—original draft. **Jesus Shrestha**: Writing—review & editing; Writing—original draft. **Mohammad Imran**: Writing—review & editing. **Nisha Panth**: Writing—review & editing. **Shakti Dhar Shukla**: Writing—review & editing. **Saurav Kumar Jha**: Writing—review & editing. **Hari Prasad Devkota**: Supervision. **Majid Ebrahimi Warkiani**: Supervision. **Sachin Kumar Singh**: Writing—review & editing; Supervision. **Md Khadem Ali**: Writing—review & editing. **Gaurav Gupta**: Writing—review & editing; Supervision. **Dinesh Kumar Chellappan**: Supervision; Writing—review & editing. **Philip M. Hansbro**: Supervision; Writing—review & editing. **Kamal Dua**: Writing ‐ review & editing; Supervision.

## FUNDING INFORMATION

The author(s) report no funding associated with this work featured in this article.

## CONFLICT OF INTEREST

The authors declare that they have no known competing financial interests or personal relationships that could have appeared to influence the work reported in this paper.

## Data Availability

Data sharing is not applicable to this article as no new data were created or analyzed in this study.
